# Mechanism of total saponins of *Ranunculus ternatus* Thunb. in treatment of breast cancer based on liquid chromatography–mass spectrometry and network analysis

**DOI:** 10.3389/fphar.2025.1506885

**Published:** 2025-04-25

**Authors:** Yuanxin Zhang, Shuo Nan, Wei Zhao, Haisheng Xie, Jinxin Miao, Mingsan Miao

**Affiliations:** ^1^ Department of Pharmacy, Henan University of Chinese Medicine, Henan, China; ^2^ Department of Academy of Chinese Medicine, Henan University of Chinese Medicine, Henan, China

**Keywords:** total saponins of *Ranunculus ternatus Thunb.*, breast cancer, inflammation, JAK2/STAT3 signaling pathway, LC-MS, network analysis

## Abstract

**Objective:**

To explore the mechanism of total saponins of *Ranunculus ternatus* Thunb. (RT) in the treatment of breast cancer (BC) using liquid chromatography–mass spectrometry (LC-MS) technology and network analysis.

**Methods:**

The metabolites of RT were detected using LC-MS. Metabolites and targets of RT and BC were identified in different databases, and potential targets and pathways were predicted using protein–protein interaction network and pathway enrichment analyses. A mouse model of BC created by cellular injection and MCF-7 cells were used as research objects for *in vivo* and *in vitro* validation experiments to study the anti-BC mechanism of RT.

**Results:**

A Kyoto Encyclopedia of Genes and Genomes analysis showed that the Janus kinase/signal transducer and activator of transcription signaling pathway might be associated with the anti-BC effects of RT. The *in vivo* and *in vitro* experiments showed that Total saponins from RT had a good anti-BC effect that can inhibit the expression of JAK2 and STAT3-related proteins and mRNA, affect the expression levels of serum inflammatory factors tumor necrosis factor-α, interleukin-6, and interleukin-10, inhibit tumor growth, proliferation, and migration, and promote tumor cell apoptosis.

**Conclusion:**

Total saponins from RT may play a role in BC treatment by regulating the JAK2/STAT3 signaling pathway.

## Graphical Abstract

**Figure:**
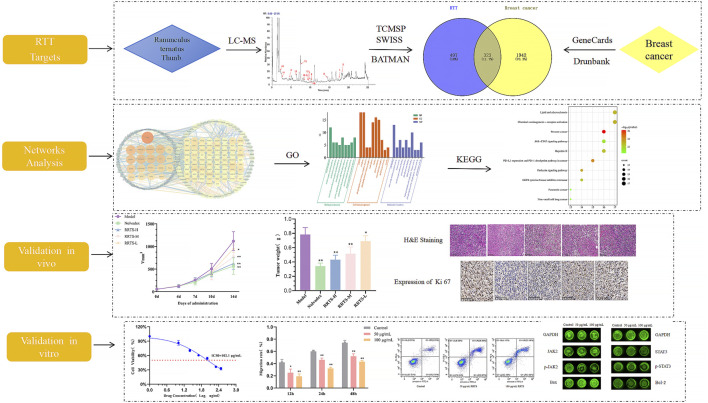
Experimental flow chart.

## 1 Introduction

Breast cancer (BC) is among the most common cancers affecting women worldwide. According to data from the International Agency for Research on Cancer of the World Health Organization, 2.3 million women will be diagnosed with BC globally in 2022, while 670,000 will die of it. Among 185 countries, BC is the most common cancer among women in 157 countries (Bray et al., 2024; Arnold et al., 2022). Risk factors for BC include advanced age, obesity, high alcohol consumption, family history of BC, history of radiation exposure, reproductive history (e.g., age at menarche and age at first pregnancy), tobacco use, and postmenopausal hormone therapy (Yu et al., 2023; Zhang et al., 2024). Mutations in certain genetic “high penetrance” genes (e.g., *BRCA1*, *BRCA2*, and *PALB-2*) significantly increase a woman’s risk of BC (Patel et al, 2024; Moar et al., 2023). Breast cancer treatment depends on the cancer subtype and extent of spread from the breast to the lymph nodes (stage II or III) or other parts of the body (stage IV). The primary treatment methods include surgery, radiation, chemotherapy, and endocrine therapy (Wu et al., 2022). These methods can significantly improve the clinical symptoms and reduce the recurrence rate of BC. However, issues such as toxic side effects and drug resistance associated with chemotherapy drugs seriously affect patient quality of life. Studies have shown that traditional Chinese herbal medicines have advantages, such as multiple pathways of action, multiple targets, multiple effects, multi-directionality, and low toxicity (Yang et al., 2021). The search for efficient anti-tumor drugs derived from natural sources has become a new trend in the field of BC research.

*Ranunculus ternatus* Thunb. (RT) is a natural anti-tumor drug belonging to the Ranunculaceae family. It is spindle-shaped and clustered, resembling a cat’s claw. RT is a medicinal plant rich in bioactive compounds, including alkaloids, polyphenols, flavonoids, triterpenoids, and polysaccharides. These constituents contribute to its diverse pharmacological properties, such as expectorant, anti-inflammatory, detoxifying, and anti-edema effects. It can be used to treat lung and breast cancers as well as lymphomas (Wang et al., 2022). Recent research has shown that the RT can effectively inhibit MCF-7 BC cell proliferation and induce their apoptosis (Yang and Zhang, 2021). RT has received widespread attention for its significant anti-tumor effects, but research on its anti-cancer mechanism and specific pharmacodynamic substances is relatively limited. Therefore, using liquid chromatography–mass spectrometry, network analysis, and experimental verification, this study analyzed the mechanism of action of RT against BC to provide a theoretical basis for its clinical application.

Network pharmacology is an emerging strategy that combines bioinformatics with traditional Chinese medicine (Xu et al., 2021). In the present study, the targets of RT and BC were identified in different databases, and the key targets and pathways of RT for treating BC were predicted using protein–protein interaction (PPI) network analysis and pathway enrichment analysis. This pathway was validated by *in vivo* and *in vitro* pharmacological experiments.

## 2 Material and methods

### 2.1 Reagents and antibodies

The following supplies were used: Eleutheroside A standard (Chengdu Pusi Biotechnology; PS0811-0025); *R. ternatus* Thunb.plant medicine (Xinyang, Henan Province); a mouse IL-6 kit (Suzhou Calvin Biotechnology CK-E20012M); a mouse TNF-α kit (CK-E20220M); a mouse IL-10 kit (CK-E20206M); a human IL-6 kit (Suzhou Calvin Biotechnology Co., Ltd.; CK-E20665M); a human TNF-α kit (CK-E20036M); a human IL-10 kit (CK-E20667M); fetal bovine serum (Ausbian; S711-001S); RPMI-1640 basic medium with dual antibiotics (Wuhan Procell Life Science & Technology; PM150110A); rabbit anti-Bax primary antibody (Beijing Bioss; bs-0127R); rabbit anti-Bcl-2 primary antibody (Beijing Bioss; bs-4563R); rabbit GAPDH (GAPDH; Wuhan Sanying Biotechnology; GB-15004); rabbit anti-p-JAK2 primary antibody (Bioss; bs-3206R); Goat Anti-Rabbit IgG H&L antibody (Bioss; bs-40295G-IRDye800CW); primer sequence information 5′–3′ primer sequence: Mouse GAPDH (Forward: CCT​CGT​CCC​GTA​GAC​AAA​ATG, Reverse: TGA​GGT​CAA​TGA​AGG​GGT​CGT); Mouse JAK2 (Forward: GTG​CTT​TTG​AAG​ACA​GGG​ACC, Reverse: GGG​TCA​TAG​CGG​CAC​ATC​TC); MouseSTAT3(Forward:TGCGGAGAAGCATTGTGAGTG, Reverse: TCT​TAA​TTT​GTT​GGC​GGG​TCT); Human GAPDH (Forward: GGA​AGC​TTG​TCA​TCA​ATG​GAA​ATC, Reverse: TGA​TGA​CCC​TTT​TGG​CTC​CC); Human JAK2 (Forward: GGC​CTT​CTT​TCA​GAG​CCA​TCA, Reverse: TTT​TAC​AGC​GAC​CAC​CTC​CC); Human STAT3 (Forward: CTC​AAC​TAT​CTG​GAG​GAC​AAA​GGC, Reverse: TGA​CGC​CAC​TAA​ACA​CTT​CCC); Human Bax (Forward: CGG​GTT​GTC​GCC​CTT​TTC​TA, Reverse: GAG​GAA​GTC​CAA​TGT​CCA​GCC); Human Bcl-2 (Forward: GGA​GGA​TTG​TGG​CCT​TCT​TTG, Reverse: GCA​TCC​CAG​CCT​CCG​TTA​TC); microplate reader (BIO-RAD, United States; BIO-RAD680).

### 2.2 Analysis of RT metabolites using LC-MS

Accurately weigh 5 g of RT, add 30 mL (6×) of 85% ethanol in a stoppered flask, and reflux for 3 h. After cooling, filter, concentrate under reduced pressure, dissolve in methanol (5 mL), and filter (0.22 μm) to obtain the test solution. Next, we dissolved 10 mg of Eleutheroside A in 10 mL of methanol to prepare an internal standard stock solution and stored it at 4°C. Before use, we vortexed the solution for 30 s, centrifuged it at 13,000 r·min^-1^ for 15 min (centrifugal radius, 13.5 cm), and collected the supernatant and filtered it through a 0.22-µm microporous membrane to obtain the internal standard solution (Bobo, 2019; Liang et al., 2023).

An Agilent SB-C18 liquid chromatography column (1.8 μm; 2.1 mm × 100 mm) was used. Mobile phase A was 0.1% formic acid in ultrapure water, while mobile phase B was 0.1% formic acid in acetonitrile. From 0 min to 9 min, the ratio of mobile phase B increased linearly from 5% to 95%. Subsequently, the ratio of mobile phase B was maintained at 95% for 10 min. From 10 to 11.10 min, the ratio of mobile phase B decreased from 95% to 5%. The ratio of mobile phase B was maintained at 5% for 14 min. The mobile phase flow rate was kept at 0.35 mL/min. The column temperature was set as 40°C. The injection volume for sample extraction was 2 µL (Fu et al., 2021).

The electrospray ionization temperature for mass spectrometry was 500°C. The spray voltage was 5500 V for the positive model and 4500 V for the negative model. Gas I of the ionization source, gas II of the ionization source, and the curtain gas were 50, 60, and 25 psi, respectively. Multiple reaction monitoring (MRM) was used for the QQQ scanning of the metabolites. Collision gas (N_2_) was used as the medium. The declustering potential and collision energy of each MRM precursor and product ion were optimized (Tian et al., 2021).

Mass spectra data, such as secondary spectra, retention time, and accurate mass of the precursor and product ions of each metabolite, were matched to the Metware database (Wuhan Metware Biotechnology). The tolerance of the secondary spectrum was set as 20 ppm, and the retention time offset was less than 0.2 min (Ren et al., 2020; Liu et al., 2020). Thus, the chemical profile of the alcohol extract of RT was determined.

### 2.3 Prediction of drug targets for RT and BC

The main chemical metabolites of RT were collected by TCM System Pharmacology (http://ibts.hkbu.edu.hk/LSP/tcmsp.php) and Swiss Target Prediction (http://swisstargetprediction.), and the compound name and molecular structure were confirmed through the PubChem database (https://pubchem.ncbi.nlm.nih.gov/). Next, collected the possible targets among the RT compounds and verified the target names by using the UniProt database (Patel and Adrada, 2024) (https://www.uniprot.org/).

### 2.4 Prediction of targets for BC

In the GeneCards database (https://www.genecards.org/), Drug Bank database (https://go.drugbank.com/) and OMIM database (https://www.omim.org/), “Breast cancer” were used as keywords to search, download, and integrate all related genes.

### 2.5 Construction of PPI network, gene ontology, and kyoto encyclopedia of genes and genomes enrichment analysis

The intersection between the RT targets and BC was used to obtain the therapeutic targets of RRT using the search term “Venny.” A network diagram of the active RRT metabolites was constructed using Cytoscape software, and then the network analysis tool in Cytoscape was used to analyze the topological parameters of each network and to evaluate the significance of the nodes according to centrality and node degree. Therapeutic targets and protein–protein interaction (PPI) networks were constructed using the STRING database (https://string-db.org/). The conditions were as follows: “minimum required interaction score = 0.9″ and “hide disconnected nodes in the network.” The David v6.8 database (https://david.ncifcrf.gov/) was used to perform Gene Ontology (GO) and Kyoto Encyclopedia of Genes and Genomes (KEGG) pathway enrichment analyses.

### 2.6 Animal experiment

*Ranunculus ternatus* Thunb. has demonstrated significant anti-tumor activity in both traditional Chinese medicine and modern research. Its metabolites have been shown to inhibit various tumor cells, including BC cells (Yin et al., 2008; Chen et al., 2016; Huanbin et al., 2022). In this study, RT metablites were selected based on LC-MS results, and related pathways were predicted based on network pharmacological analysis, and finally preliminarily verified through *in vitro* and *in vivo* experiments.

Eighty SPF-grade BALB/c mice (female, 8 weeks, 18 ± 2 g, Animal license number: 370726231100817586, Ethics Committee of Henan University of Chinese Medicine Ethics lot number DWLLGZR202202217.) were purchased from Jinan Pengyue Experimental Animal Breeding,which Co., LTD., license number is SCXK(Lu)20220006. The experimental mice were reared in the barrier system of the Laboratory Animal Center of Henan University of Traditional Chinese Medicine, License No. SYXK(Henan)2022-0015.

Twelve mice were randomly selected for the blank group, and the remaining mice were used as the transplanted BC mouse model. Murine 4T1 cells in the logarithmic growth stage were collected and centrifuged in a centrifuge tube at 1100 rpm for 3 min, the supernatant was discarded, and the cell suspension concentration was adjusted to 1 × 10^7^ cells/mL after being suspended in phosphate-buffered saline (PBS). The cells were injected *in situ* into the fat pad below each mouse’s fourth pair of mammary glands; 0.2 mL of the cell suspension was inoculated subcutaneously (2 × 10^6^ cells total) (Yang et al., 2020). After the cells were inoculated, the mice were observed daily until the tumor size reached approximately 50 mm^3^ (excluding cases with larger or smaller tumors) (Katuwal et al., 2023). The mice were randomly divided into model, Nolvadex (3.4 mg/kg/d), high, medium and low dose groups of the RT metabolites, and were administrated intragastric.Samples were taken after the last administration.

#### 2.6.1 Tumor growth observation

From the first day of administration, the long (a) and short (b) diameters of the tumors were measured with Vernier calipers every 3 days, and the tumor volume was calculated according to the following formula: tumor volume (mm^3^) = (a × b^2^)/2 (Fang et al., 2022). Two hours after the last administration, kill the mouse and collect blood from the inner canthus of the eye, then the tumor tissue was removed, and the tumor was weighed.

#### 2.6.2 Changes in serum levels of inflammatory factors

Blood was collected from the mice and centrifuged at 3500 r/min for 15 min. The expression levels of serum inflammatory cytokines IL-10, IL-6, and TNF-α were detected using an enzyme-linked immunosorbent assay (ELISA) kit.

#### 2.6.3 Histopathology

The tumor tissues were fixed in 4% paraformaldehyde and embedded in paraffin. Tumor sections (5 μm thick) were stained with hematoxylin and eosin.

#### 2.6.4 Immunohistochemistry

Paraffin sections of the tumor tissue were broiled, dewaxed with xylene, and subjected to gradient ethanol hydration, antigen repair, removal of endogenous peroxidase, and 5% bovine serum albumin closure. The primary antibody was incubated at 4°C overnight, horseradish peroxidase–labeled goat anti-rabbit immunoglobulin G and incubated at 37°C for 30 min. After 3,3ʹ-diaminobenzidine color development, hematoxylin redyeing, and other steps, the film was sealed and microscopy was performed. Finally, ImageJ software was used to perform a semi-quantitative analysis.

#### 2.6.5 Quantitative real-time polymerase chain reaction

Total RNA was extracted from the tumor tissue using the FastPure Cell/Tissue Total RNA Isolation Kit V2 (Nanjing Vazyme Biotech, RC112),and the tested RNA was reverse-transcribed into cDNA using the HiScript III RT SuperMix for qPCR (+ gDNA wiper) (Nanjing Vazyme Biotech, R323). A ChamQ Universal SYBR qPCR Master Mix kit (Nanjing Vazyme Biotech, Q711) was used to perform the reactions using a fluorescent quantitative real-time polymerase chain reaction (qRT-PCR) instrument. Product specificity was determined using a melting curve after each cycle. The expression was calculated using the 2^−ΔΔCT^ method. Primers were synthesized by SERVICEBIO Biotechnology Company (Wuhan, China).

### 2.7 Cell experiments

#### 2.7.1 Cell culture and viability assay

Human BC MCF-7 cells previously cryopreserved by our research group were identified as human BC cells via short tandem repeat analysis without contamination with *mycoplasma* or *chlamydia*. The cells were cultured in RPMI-1640 medium containing 10% fetal bovine serum and 1% dual antibiotics (complete medium). The incubator was set at 37°C with a CO_2_ concentration of 5%.

Log-phase MCF-7 cells were harvested, the old medium was discarded, and the cells were washed twice with PBS. The cells were digested with trypsin, collected by centrifugation, resuspended, and counted. We adjusted the concentration of the MCF-7 cells to 1.0 × 10^5^ cells/mL using complete medium and seeded them into 96-well plates at 100 μL/well. After 24 h of culture, the cells were treated with complete media containing different concentrations of RT metabolites. Complete medium was added to the blank wells; complete medium and cells were added to the control wells; and complete medium, cells, and drug were added to the experimental wells. After 24 h of incubation, the medium was replaced and 10 μL of CCK-8 was added to each well. After incubation for 2 h, absorbance was measured at 450 nm. Cell viability and half-maximal inhibitory concentration (IC_50_) were calculated based on the absorbance values (Zhai et al., 2022).

#### 2.7.2 Cell scratch assay

A cell scratch healing assay was also performed (Ruan et al., 2023). MCF-7 cells (2 × 10^5^ cells/well) were seeded into six-well plates and divided into MCF-7 control and Drug group containing RT metabolites groups. After 24 h, when the cells exceeded 90% confluence, a scratch was made vertically using a 20 μL pipette tip. The detached cells were washed three times with sterile PBS and the medium was replaced with serum-free medium. The plates were incubated at 37°C in a CO_2_ incubator. The cells were observed and photographed under a microscope at 0, 12, 24, and 48 h. The scratch area was analyzed using ImageJ software, and the migration rate was calculated as follows: migration rate (%) = [(scratch area at 0 h - scratch area at different time points)/scratch area at 0 h] × 100%.

#### 2.7.3 Cell apoptosis

The cells were seeded into six-well plates at a density of 2 × 10^5^ cells/well. Once the cells were completely adherent, they were divided into control and metabolites group. After 24 h of drug treatment, the cells were collected and centrifuged at 1200 r/min for 5 min. The cells were washed twice with PBS, resuspended in binding buffer, and stained with Annexin V-FITC/PI. After being mixed, the cells were incubated in the dark for 20 min, diluted with binding buffer, and analyzed using flow cytometry.

#### 2.7.4 ELISA assay

The cell concentration was adjusted to 1.0 × 10^5^ cells/mL and seeded into 96-well plates at 100 μL/well. The cells were divided into control and metabolites group. After 24 h of drug treatment, the supernatants were collected and centrifuged at 3500 r/min for 15 min, and the filtrates were collected and aliquoted. The levels of the inflammatory factors IL-10, IL-6, and TNF-α in the supernatants were measured using ELISA kits.

#### 2.7.5 In cell western

The concentration of MCF-7 cells was adjusted to 1 × 10^5^ cells/mL and the cells were seeded into 96-well plates at 100 μL/well. The samples were divided into control and metabolites group. After 24 h of drug treatment, the medium was discarded and in-cell Western blotting was performed to measure the proteins expression levels (Kan et al., 2024). The specific steps were as follows:

(1) Cell fixation: Add 150 μL of 4% cell/tissue fixation solution, incubate at room temperature for 20 min and avoid shaking.
(2) Cell permeabilization: Discard the fixation solution, add 200 μL of 0.1% Triton-X-100 elution buffer, gently shake at room temperature for 5 min, and repeat five times.
(3) Blocking: After elution, discard the 0.1% Triton-X-100 elution buffer, add 150 μL of 5% skim milk blocking solution, incubate at room temperature for 1 h, and recover the blocking solution.
(4) Primary antibody incubation: Add 50 μL of primary antibody (prediction pathway of network pharmacological analysis)and internal control incubate overnight at 4°C, recover the primary antibody, and wash with 0.1% Tween-20 in PBS five times for 5 min each.
(5) Secondary antibody incubation: Add 50 μL of Goat Anti-Rabbit IgG H&L antibody (1:500), incubate it in the dark at room temperature for 1 h, recover the secondary antibody, and wash with 0.1% Tween-20 in PBS five times for 5 min each.
(6) Imaging: Immediately scan the microplate using a near-infrared dual-color imaging system.

#### 2.7.6 Quantitative real-time polymerase chain reaction

Total RNA was extracted from the control and experimental cells.It was reverse-transcribed into cDNA and stored at −80°C. Finally, qRT-PCR was performed using ChamQ Universal SYBR qPCR Master Mix and relative expression calculated using the 2^−ΔΔCt^ method.

### 2.8 Statistical analysis

All data are expressed as mean ± standard deviation. The significance of the intergroup differences was analyzed by one-way analysis of variance, followed by Dunnett’s multiple comparison test, using GraphPad Prism. Values of *P* < 0.05 were considered statistically significant.

## 3 Results

### 3.1 Identification of RC metabolites

**FIGURE 1:**
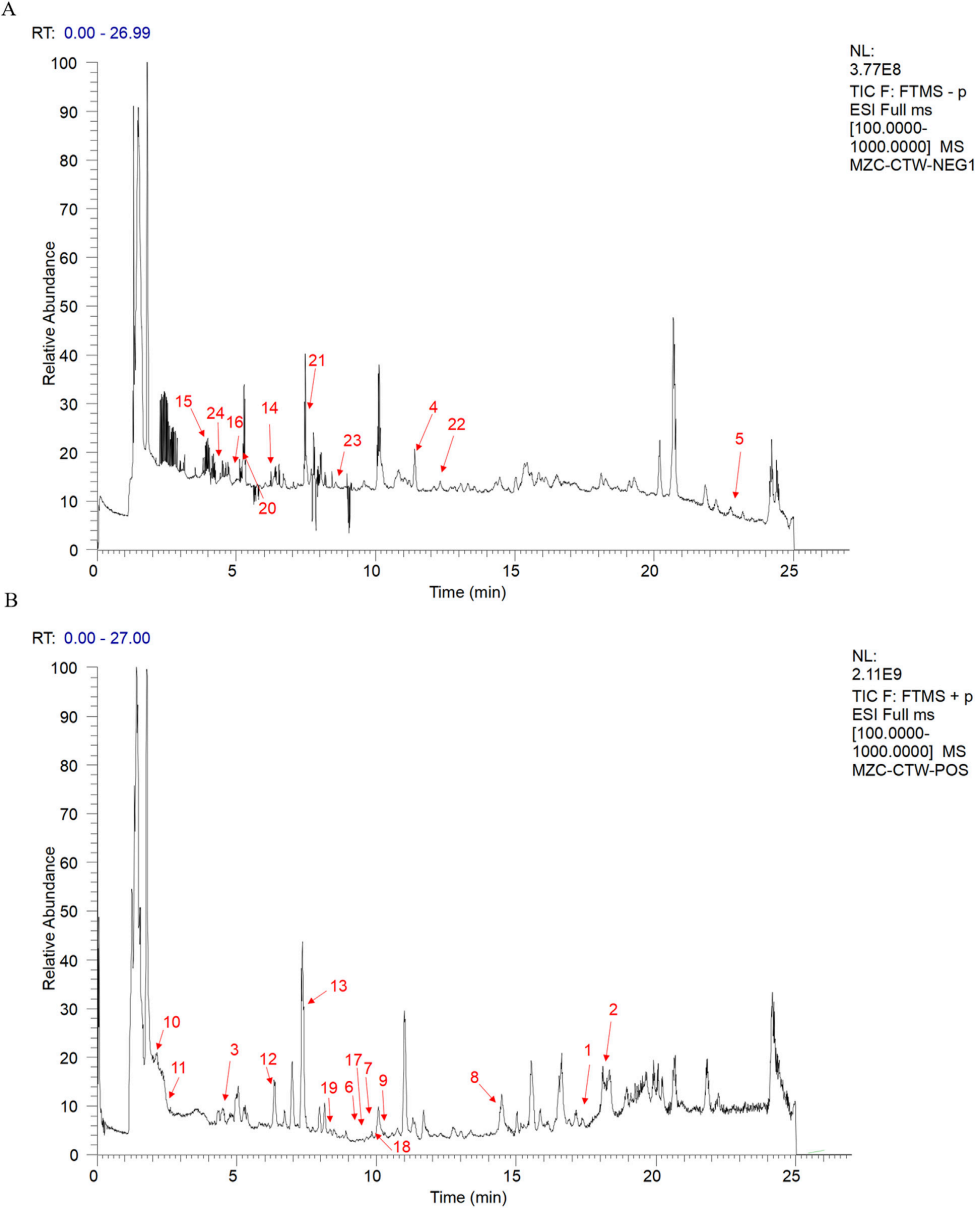
Positive and negative ion spectra of *Ranunculus ternatus* Thunb. Metabolites [**(A)** anion **(B)** Positive ion The numbers 1-24 in the figure represent the 24 Terpenoids and their derivatives].

Phytochemical analysis identified 53 compounds which were classified into three major categories: terpenoids (24 compounds, including 5 characteristic triterpenoid saponins with glycosylation modifications), phenolics (17 flavonoids and phenolic acids), and special structural compounds (12 components such as HMF and phthalides) (Supplementary Table S2; Figure 1). Notably, terpenoids constituted the predominant chemical class (45.3%), with triterpenoid saponins (e.g., phytolaccagenin, asiatic acid, and madecassic acid) demonstrating particularly high abundance, as reflected by their superior chromatographic responses (intensity values reaching 508,000) compared to other compound classes. These saponins exhibited well-defined peaks in the mid-to-late retention time window (15–23 min), suggesting their significant accumulation in the extract. Literature supports that saponins represent structurally unique plant secondary metabolites that frequently serve as key pharmacologically active components, possessing well-documented anti-inflammatory and anticancer properties (Xia et al., 2023; Xiang et al., 2025; Yin et al., 2021). Based on these phytochemical characteristics and established bioactivities, we selected the total saponins from *R. ternatus* Thunb.(RRTS) as our research focus to systematically investigate their potential anti-breast cancer mechanisms.

### 3.2 Identification of RT-related targets and BC-related targets

Using the TCMSP and Swiss Target Prediction databases, 56 active metabolites and 821 targets of RRT were screened based on the presence or absence of targets. BC targets were obtained from the GeneCards and DrugBank databases. After removing duplicates and using the MEDIAN function three times on the relevance score column, 2311 targets were screened.

### 3.3 Construction of RRT-BC intersection targets network

**FIGURE 2:**
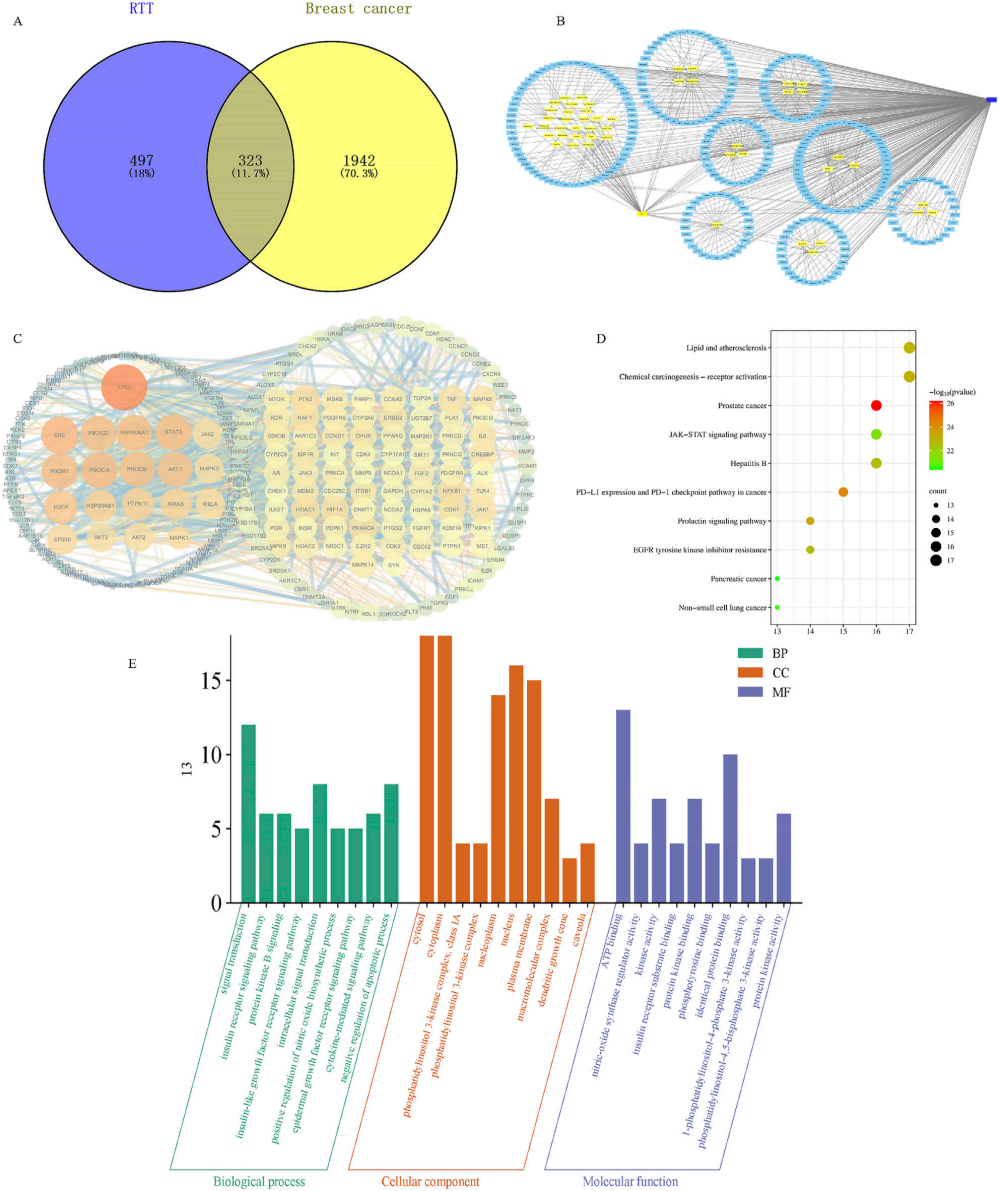
Results of network pharmacological analysis of *Ranunculus ternatus* Thunb. **(A)** Venn diagram of active ingredients and disease targets. **(B)** RT active ingredient and breast cancer-target network. **(C)** Network of the main targets of RT treatment of breast cancer. **(D)** Analysis of KEGG pathway enrichment. **(E)** GO functional enrichment analysis.

Based on the Venn diagram, the intersection of RRT target genes and BC disease targets identified as key targets comprised 323 targets (Figure 2A). Further, the “Traditional Chinese Medicine-metabolites-Target” network for RRT in the treatment of BC was constructed using Cytoscape visualization software (Figure 2B). A PPI network diagram was created using the STRING 11.0 database (https://string-db.org/) and imported into Cytoscape for visualization. In the diagram, nodes represent proteins and edges represent the interactions between proteins. The larger and darker the node, the more important it is as indicated by degree value (Wang et al., 2024). The top 20 targets, ranked by degree values, were selected as core targets and included TP53, SRC, PIK3CA, PIK3R1, HSP90AA1, STAT3, PIK3CD, PIK3CB, AKT1, EGFR, HSP90AB1, PTPN11, NRAS, EP300, MAPK1, JAK2, MAPK3, AKT2, AKT3, and RELA (Figure 2C).

### 3.4 GO and KEGG analysis of JB-RA intersection targets

After the GO enrichment analysis, 222 biological processes were identified, including phosphorylation, signal transduction, the insulin receptor signaling pathway, protein kinase B signaling, and the insulin-like growth factor receptor signaling pathway. A total of 27 cellular metabolites were obtained, including the cytosol, cytoplasm, plasma membrane, macromolecular complexes, and nucleus. Fifty molecular functions were identified, including ATP binding, nitric oxide synthase regulator activity, kinase activity, insulin receptor substrate binding, and protein kinase binding processes are shown in Figure 2E. The KEGG enrichment analysis revealed 141 potential pathways, and the 10 most significantly enriched pathways (Figure 2D) included prostate cancer, Janus kinase/signal transducer and activator of transcription (JAK/STAT) signaling, non-small cell lung cancer, prolactin signaling, lipid, and atherosclerosis (Liu et al., 2023).

According to the results of network pharmacological analysis, both JAK2 and STAT3 ranked among the 20 core targets with the highest connectivity, suggesting that JAK2 and STAT3 played a key role in the pharmacological action of RT. The results of pathway enrichment analysis showed that the JAK/STAT signaling pathway was one of the most significantly enriched pathways. In addition, a large number of studies have confirmed that JAK2/STAT3 signaling plays an important role in breast cancer progression, regulating key processes including cell proliferation, survival and immune escape (Jiang et al., 2024; Li et al., 2023; Mengie et al., 2022). At the same time, the JAK/STAT pathway interacts with other significantly enriched cancer-related pathways, such as the prolactin signaling pathway, suggesting that it may act as a key regulatory hub. These comprehensive findings provide a sufficient theoretical basis for further experimental verification of the role of JAK2/STAT3 axis in the anti-breast cancer mechanism of RT.

### 3.5 Animal experiment

We selected an appropriate amount of dried RC, crushed it into coarse particles, and added six times the amount of 85% ethanol. The samples were extracted for 3 h and then filtered. Ethanol was evaporated from the filtrate, extracted twice with petroleum ether, and extracted three times with water-saturated n-butanol. The n-butanol solution was extracted twice with 0.1 mol/L NaOH and evaporated to obtain the RRTS.The mice were randomly divided into control, Nolvadex (3.4 mg/kg/d), RRTS high-dose (RRTS-H; 200 mg/kg/d), RRTS medium-dose (RRTS-M; 100 mg/kg/d), and RRTS low-dose (RRTS-L; 50 mg/kg/d) groups. The treatments (0.1 mL/10 g) were administered by gavage. Twelve mice from each group were subjected to continuous intragastric administration for 14 days. Mice in the blank group were administered an equal volume of normal saline solution. Two hours after the last administration, the samples were collected and their indices detected.

#### 3.5.1 Effects of RRTS on tumor volume and weight in mice with transplanted BC

**FIGURE 3:**
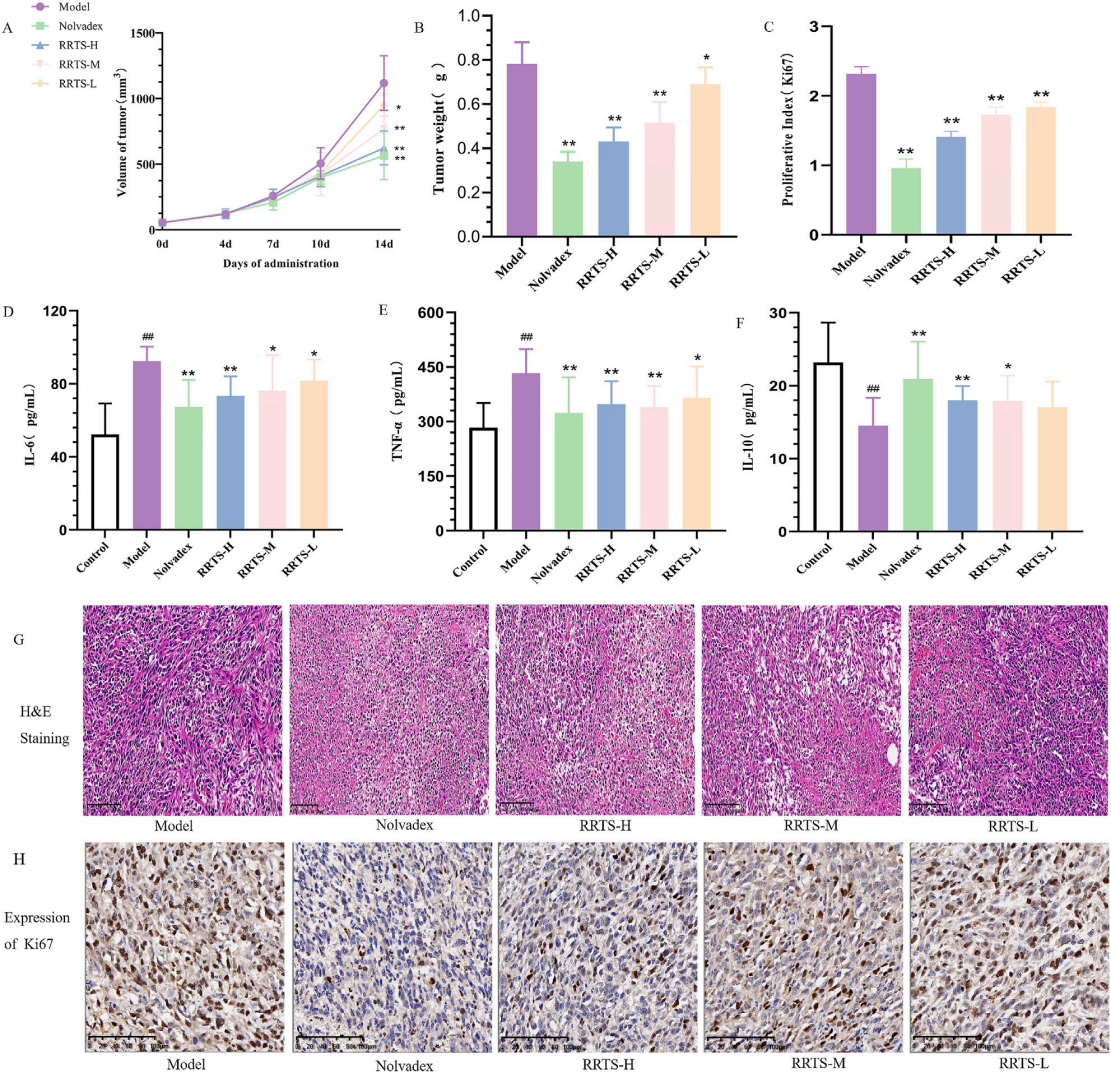
Effect of RRTS on the growth of breast cancer mice. **(A, B)** Effects on tumor volume and weight in mice with breast cancer. **(D, E)** Effects on inflammatory cytokines IL-6, TNF-α and IL-10. **(F)** Effect on tumor histopathology in mice. **(C, G–H)** Effect on Ki67 protein expression in tumor tissue. Data were shown as the mean ± SD of three independent experiments. Compared with control group, *P < 0.05, **P < 0.01.

On day 0 of administration, the tumor volumes in the model and treatment groups were similar (*P* > 0.05), indicating uniform grouping. On day 4 of administration, the tumor volumes in each treatment group were similar to those in the model group, with no significant improvement. On day 7 of administration, the tumor volume was significantly lower in the Nolvadex than model group (*P* < 0.05). The other treatment groups showed varying degrees of tumor volume reduction, but the differences were not significant (*P* > 0.05). On day 10 of administration, compared with the model group, the Nolvadex group showed a significant reduction in tumor volume (*P* < 0.01), while the RRTS-H group showed a significant reduction in tumor volume (*P* < 0.05). The other treatment groups showed a decreasing trend in tumor volume, but the difference was not significant (*P* > 0.05). On day 14 of administration, compared to the model group, the tumor volume in the Nolvadex, RRTS-H, and RRTS-M treatment groups was significantly decreased (*P* < 0.01), while the tumor volume in the RRTS-L group was significantly reduced (*P* < 0.05; Figure 3A). Compared to the model group, the tumor weights in the Nolvadex, RRTS-H, and RRTS-M treatment groups were significantly decreased (*P* < 0.01), while the tumor weight in the RRTS-L group was significantly reduced (*P* < 0.05; Figure 3B).

#### 3.5.2 Effects of RT on serum inflammatory factors in mice with transplanted BC

The levels of serum inflammatory factors TNF-α, IL-6, and IL-10 in the mice were calculated and analyzed according to the instructions of the assay kits. As shown in Figures 3D–F, compared to the control group, the mean serum IL-6 level in the model group was significantly higher (*P* < 0.01). Compared to the model group, the serum IL-6 levels in the Nolvadex and RRTS-H groups were significantly decreased (*P* < 0.01), whereas the IL-6 levels in the RRTS-M and RRTS-L groups were notably decreased (*P* < 0.05). Compared to the control group, the serum TNF-α level in the model group was significantly increased (*P* < 0.01). Compared to the model group, the serum TNF-α levels in the Nolvadex, RRTS-H, and RRTS-M groups were significantly decreased (*P* < 0.01), while the TNF-α level in the RRTS-L group was notably decreased (*P* < 0.05). Compared to the control group, the serum IL-10 level in the model group was significantly lower (*P* < 0.01). Compared to the model group, the serum IL-10 levels in the Nolvadex and RRTS-H groups were significantly increased (*P* < 0.01), whereas the IL-10 level in the RRTS-M group was notably increased (*P* < 0.05). The IL-10 levels in the RRTS-L group showed an increasing trend.

#### 3.5.3 Effects of RT on pathological changes and Ki-67 proliferation protein in tumor tissue of mice with transplanted BC

The effects on the pathology of mouse tumor tissues are shown in Figure 3G. In the model group, the tumor tissues contained abundant tumor cells, a disordered and dense arrangement, and large areas of nuclear pyknosis (Zhang et al., 2023). The tumor cells had small intercellular spaces that formed clusters or cords. In the Nolvadex group, the number of tumor cells was significantly reduced with a relatively sparse cell arrangement and clear morphology. Compared with the model group, the number of tumor cells in the RRTS-treated groups decreased, and the cell arrangement became more orderly and distinct. The tumor tissue of the mice continued to increase as the dose of RC total saponins increased.

Ki-67 protein is a reliable marker for detecting tumor cell proliferation activity (Li et al., 2021). Immunohistochemistry experiments revealed that, compared to the model group, the expression of Ki-67 protein in the tumor tissues of the Nolvadex- and RRTS-treated groups was significantly reduced (*P* < 0.01; Figures 3C, H). These results indicate that both the positive control (Nolvadex) and RT can inhibit tumor growth in mice with BC.

#### 3.5.4 Effects on JAK2, p-JAK2, STAT3, and p-STAT3 protein expression in tumor tissue of mice with transplanted BC

**FIGURE 4:**
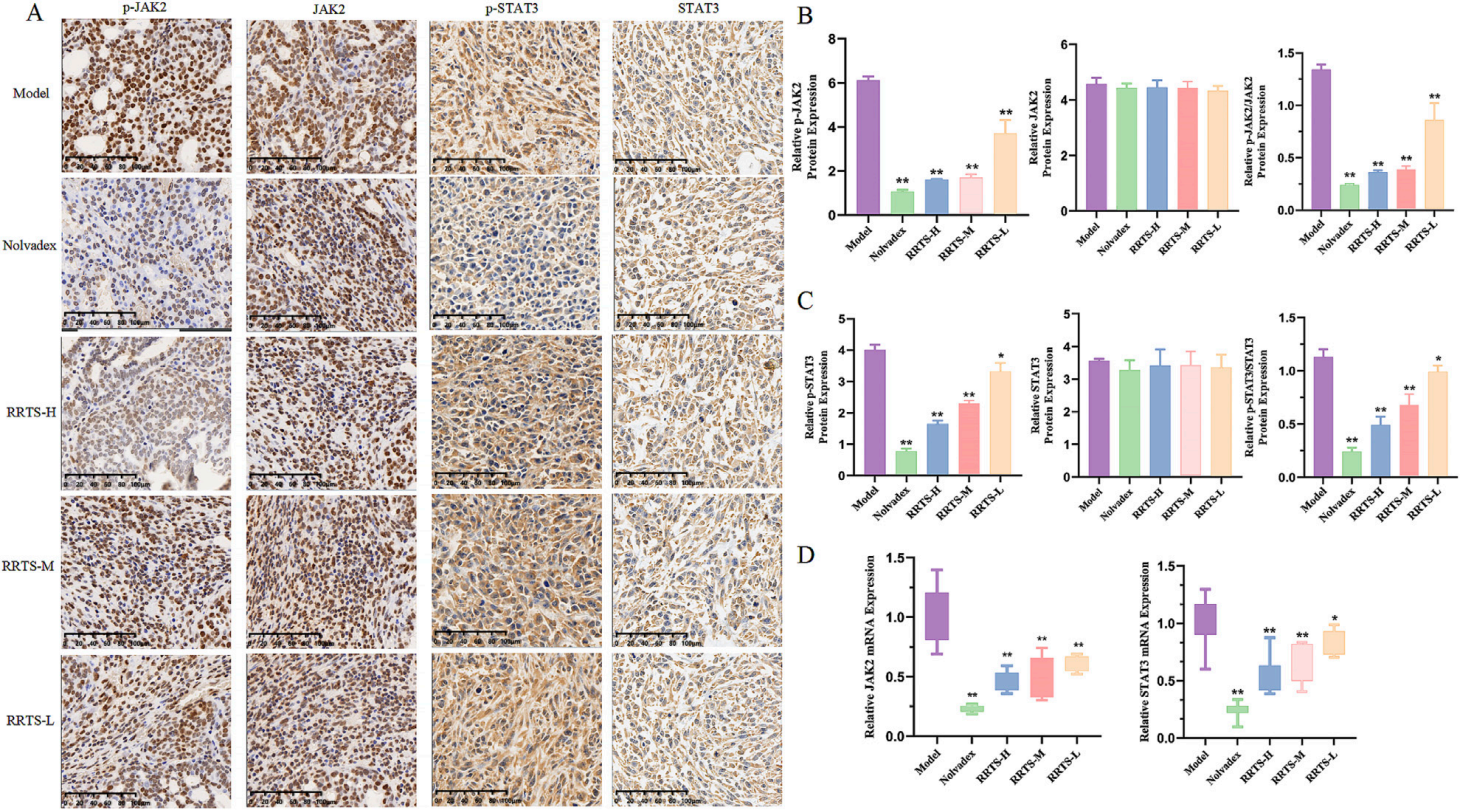
Effect of RRTS on JAK2/STAT3 pathway. **(A–C)** Relative protein expression of p-JAK2, p-STAT3, JAK2, and STAT3. **(D)** mRNA expression of JAK2 and STAT3. Data were shown as the mean ± SD of three independent experiments. Compared with control group, *P < 0.05, **P < 0.01.

The KEGG pathway prediction results indicated that RT might exert its anti-BC effects through the JAK2/STAT3 pathway. This was validated by immunohistochemistry, which showed that the relative expression levels of the p-JAK2 and p-STAT3 proteins were highest in the tumor tissues of the model group. Compared to the model group, the expression levels of p-JAK2/JAK2 proteins in the tumor tissues of the Nolvadex group and all RT treatment groups were significantly lower (*P* < 0.01). Compared to the model group, the expression levels of p-STAT3/STAT3 proteins in the tumor tissues of the Nolvadex, RRTS-H, and RRTS-M groups were significantly reduced (*P* < 0.01), while those in the RRTS-L group was notably reduced (*P* < 0.05; Figures 4A–C).

#### 3.5.5 Effects on JAK2 and STAT3 mRNA expression in tumor tissue of mice with transplanted BC

The qRT-PCR results are shown in Figure 4D. Compared to the model group, the mRNA expression levels of JAK2 and STAT3 in the tumor tissues of the Nolvadex group were significantly lower (*P* < 0.01). The mRNA expression levels of JAK2 in the tumor tissues of the different RRTS treatment groups were significantly reduced (*P* < 0.01). The mRNA expression levels of STAT3 in the tumor tissues of the RRTS-H and RRTS-M groups were significantly reduced (*P* < 0.01), and the expression levels in the RRTS-L group were notably reduced (*P* < 0.05).

### 3.6 Cell experiments

#### 3.6.1 Effects of RT on growth state of MCF-7 cells

**FIGURE 5:**
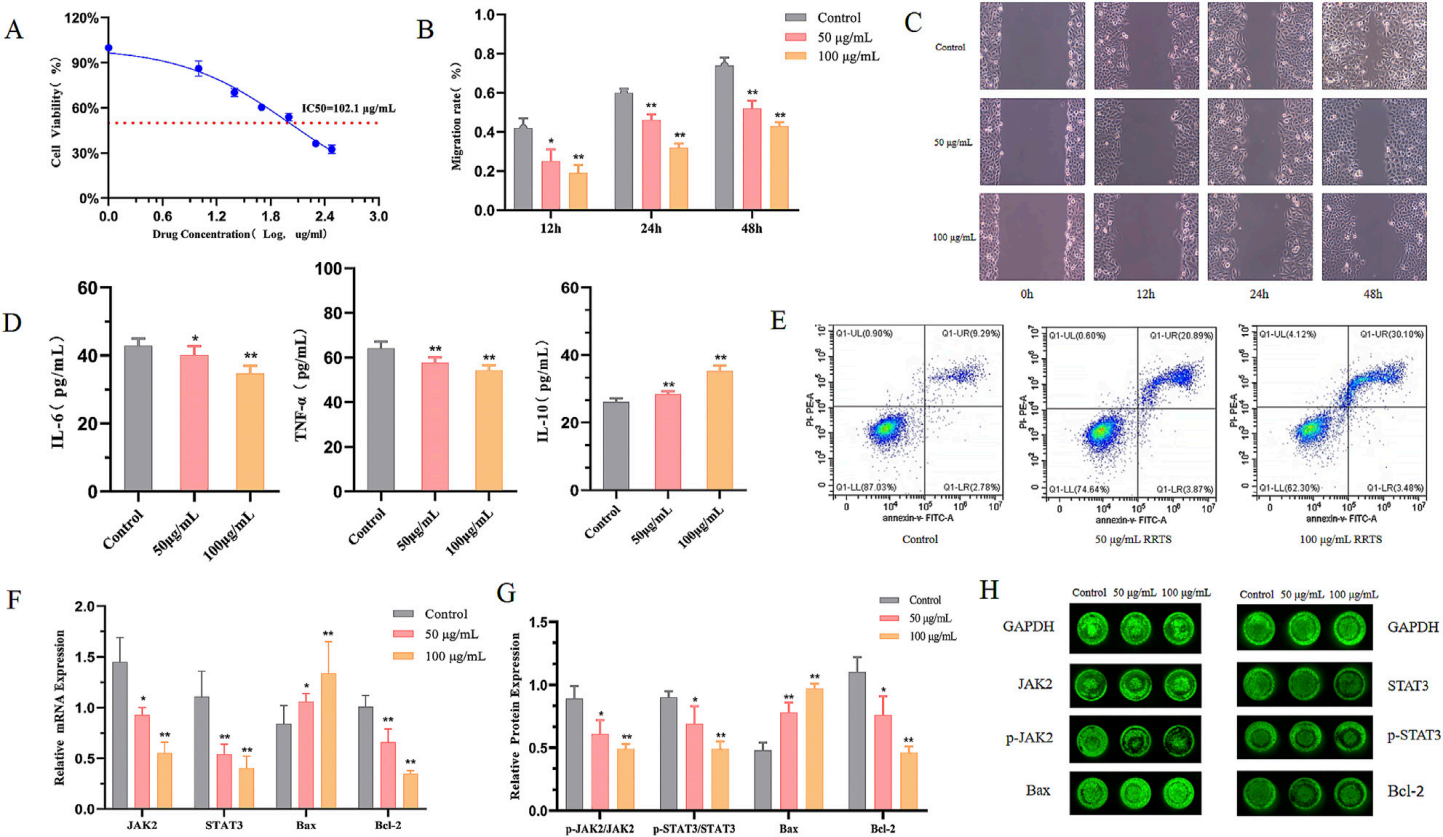
Effect of RRTS on the growth of MCF-7 cell **(A)** Effect on proliferation of MCF-7 cells. **(B, C)** Effect on MCF-7 cell migration. **(D)** Effects on inflammatory cytokines IL-6,TNF-α and IL-10. **(E)**The apoptosis rate of the cells was detected by flow cytometry (Annexin V-FITC/PI method). **(F)** mRNA expression of JAK2 and STAT3. **(G, H)** Protein expression of proteins Bax, Bcl-2, JAK2, p-JAK2, STAT3 and p-STAT3. Data were shown as the mean ± SD of three independent experiments. Compared with control group, *P < 0.05, **P < 0.01.

As shown in Figure 5A, different doses of RRTS (10, 25, 50, 100, 200, 300 μg/mL) inhibited MCF-7 cell proliferation to varying degrees. As the drug concentration increased, cell viability decreased and the inhibitory effect became more pronounced, showing a dose-dependent relationship. The IC_50_ value was 102.1 μg/mL. Therefore, we chose 50 μg/mL and 100 μg/mL as the optimal concentrations of RRTS for treating MCF-7 cells.

#### 3.6.2 Cell scratch assay

The scratch assay results are shown in Figures 5B, C. The migration area of MCF-7 cells treated with 50 μg/mL and 100 μg/mL RT for 24 and 48 h was significantly smaller than that of the control group (*P* < 0.01). The migration area of MCF-7 cells treated with 50 μg/mL RRTS for 12 h was significantly smaller than that of the control group (*P* < 0.05); for the 100 μg/mL RRTS group, the migration area was significantly smaller than that of the control group (*P* < 0.01). These results indicated that RC total saponins inhibited MCF-7 cell migration (Jia et al., 2021).

#### 3.6.3 Effects on inflammatory factors in MCF-7 cell supernatant

The cell supernatant was collected, and the levels of IL-6, IL-10, and TNF-α were measured using ELISA kits. The results (Figure 5D) showed that, compared to the MCF-7 cell control group, the expression level of IL-6 in the 50 μg/mL RRTS group was significantly reduced (*P* < 0.05), while the expression level of TNF-α was significantly reduced (*P* < 0.01). In the 100 μg/mL RRTS group, the expression levels of IL-6 and TNF-α were both significantly reduced (*P* < 0.01). Compared to the control group, the expression level of IL-10 in the 50 μg/mL and 100 μg/mL RRTS groups was significantly increased (*P* < 0.01).

#### 3.6.4 Cell apoptosis

After 24 h of RT treatment, flow cytometry results (Figure 5E) showed that, compared to the MCF-7 cell control group, the 50 μg/mL and 100 μg/mL RRTS groups significantly promoted MCF-7 cell apoptosis (*P* < 0.01) (Mirmalek et al., 2020).

#### 3.6.5 In cell western

The expression levels of p-JAK2, p-STAT3, Bax, and Bcl-2 proteins in MCF-7 cells were detected using In Cell Western. The results (Figures 5G, H) showed that, compared to the MCF-7 cell control group, the expression levels of p-JAK2/JAK2 and p-STAT3/STAT3 proteins were significantly reduced in the RRTS 50 μg/mL group (*P* < 0.05). In the RT 100 μg/mL group, the expression levels of p-JAK2/JAK2 and p-STAT3/STAT3 proteins were significantly reduced (*P* < 0.01). The expression levels of Bax protein were significantly increased in the 50 μg/mL and 100 μg/mL RRTS groups (*P* < 0.01). Compared to the control group, Bcl-2 protein expression in the RRTS 50 μg/mL group was significantly reduced (*P* < 0.05); in the RRTS 100 μg/mL group, it was significantly reduced (*P* < 0.01) (Lu and Salvino, 2023).

#### 3.6.6 Effects on JAK2, STAT3, bax, and Bcl-2 mRNA expression in MCF-7 cells

Bcl-2 and Bax are classic molecules that influence cellular proliferation and apoptosis and play important roles in tumor development. The qRT-PCR results (Figure 5F) showed that RT regulated the expression of JAK2, STAT3, and the apoptotic proteins Bax and Bcl-2 in MCF-7 cells. Compared to the control group, the mRNA expression levels of STAT3 and Bcl-2 were significantly reduced in the experimental groups (*P* < 0.01), with a greater reduction seen in the 100 μg/mL *versus* 50 μg/mL RRTS group. Additionally, compared to the control group, the mRNA expression level of JAK2 was significantly reduced in the 50 μg/mL RRTS group (*P* < 0.05) and significantly reduced in the 100 μg/mL RRTS group (*P* < 0.01). The mRNA expression level of Bax was significantly increased in the 50 μg/mL RRTS group (*P* < 0.05) and significantly increased in the 100 μg/mL RRTS group (*P* < 0.01).

## 4 Discussion

With biotechnological advances, research on BC treatment using traditional Chinese medicine has reached the molecular level. Metabolites such as traditional Chinese medicine decoctions, injections, and effective ingredients (baicalin, cucurbitacin, Astragalus polysaccharide, ginsenosides, etc.) can induce cancer cell apoptosis (Kong et al., 2021; Li et al., 2023a; Li et al., 2023b; Fan et al., 2022), affect autophagy and anti-tumor microenvironment angiogenesis, and reduce inflammatory responses. BC is an inflammation-associated tumor, and the activation of inflammatory signaling pathways is a main mechanism of breast carcinogenesis (You et al., 2023). Our results indicated that RT exerts anti-BC effects in BC mice and MCF-7 cells by inhibiting the JAK2/STAT3 inflammatory pathway.

This study employed an integrated strategy to investigate the anti-breast cancer mechanisms of Ranunculus ternatus (RT). Initial phytochemical characterization using HPLC-MS identified 53 bioactive metabolites in RT extracts. Based on their abundance and known pharmacological activities, we focused on the total saponin fraction (RTTS) for subsequent mechanistic studies. Network analysis predicted the JAK2/STAT3 signaling pathway as a potential therapeutic target of RTTS. To experimentally validate this computational prediction, we first employed an orthotopic breast cancer mouse model to assess the anti-tumor effects of RTTS *in vivo*. Dynamic monitoring of subcutaneous tumor volume changes revealed that the tumors in the model group grew rapidly, especially in the later stages, with the largest volume and heaviest tumor weight. After hematoxylin and eosin staining and microscopic observation revealed that the tumor cells were dense with small intercellular spaces, low differentiation, and large deeply stained nuclei. Ki-67 was used to assess cellular proliferation. RRTS effectively inhibited Ki-67 expression in transplanted mouse tumors. Different concentrations of RRTS had varying effects on p-JAK2 and p-STAT3 indicators in BC model mice, showing a dose-dependent relationship. Low doses of RRTS had a smaller inhibitory effect on the phosphorylation indicators of JAK2, STAT3, and their mRNA expression, whereas medium and high doses gradually increased the inhibitory effect on these indicators. IL-6, TNF-α, and IL-10 play upstream activation and downstream regulatory roles in the JAK2/STAT3 inflammatory pathway, reflecting its activation (He et al., 2024; Huang et al., 2022; Yin et al., 2018; Liu et al., 2020). Serum IL-6 and TNF-α levels were significantly elevated in BC model mice, while IL-10 levels were significantly reduced. The levels of IL-6 and TNF-α in the Nolvadex group and different dose groups of RRTS were reduced to varying degrees, while the IL-10 levels were increased. The results of animal experiments suggested that RRTS can inhibit activation of the JAK2/STAT3 inflammatory pathway, affect the expression levels of inflammatory factors TNF-α, IL-6, and IL-10 in the blood circulation, and reduce the inflammatory response in BC model mice, thereby inhibiting the malignant progression of BC.

To further clarify the anti-BC pharmacological mechanism of the RRTS based on the JAK2/STAT3 pathway, we conducted *in vitro* experiments using human-derived MCF-7 BC cells. The optimal concentrations of RRTS were determined as 50 μg/mL and 100 μg/mL by CCK-8 assay. Subsequent tests on cancer cell proliferation, growth, and apoptosis showed that the treatment of MCF-7 cells with RRTS inhibited proliferation and migration and promoted apoptosis (Dai et al., 2024). Changes in inflammatory factors in the cell supernatant were also detected, showing that the TNF-α and IL-6 levels in the drug-treated group were reduced compared to those of the blank group of MCF-7 cells, whereas the IL-10 level was significantly increased. The Western blotting results indicated that the expression levels of p-JAK2/JAK2 and p-STAT3/STAT3 in MCF-7 cells were reduced after treatment with RRTS. The qRT-PCR results showed that the mRNA levels of JAK2 and STAT3 were reduced in MCF-7 cells after drug treatment, while the mRNA level of the proliferation protein Bcl-2 was reduced and the mRNA level of the apoptosis factor Bax was increased.

**FIGURE 6:**
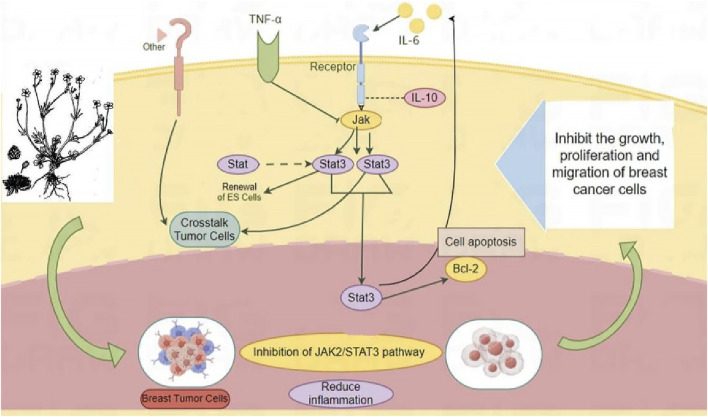
JAK2/STAT3 pathway inhibition as potential mechanism of *Ranunculus ternatus* Thunb. For the treatment of breast cancer.

The *in vivo* and *in vitro* experiments indicated that RRTS have a good anti-BC effect and that their pharmacological mechanism is related to the JAK2/STAT3 inflammatory pathway. They can inhibit JAK2 and STAT3–related protein and mRNA expression; regulate upstream and downstream pathway indicators; inhibit tumor growth, proliferation, and migration; promote tumor cell apoptosis; and reduce the inflammatory response, thereby exerting anti-BC effects (Figure 6).

## 5 Limitations

This study has several limitations that should be considered: (1) Unidentified active saponins: While total saponins showed anti-cancer effects, the specific active compounds remain unknown. Further LC-MS/MS-based structural identification and quantification are needed to clarify the active compounds. (2) Limited Scope of Bioactive Components: Our investigation concentrated solely on saponins, potentially overlooking other pharmacologically active constituents (e.g., polysaccharides, flavonoids) present in Ranunculus ternatus. (3) Overly Simplified Network Pharmacology Analysis: The current target-pathway analysis relied heavily on database predictions (e.g., KEGG, GO) Deeper investigations—such as molecular docking, protein-binding assays, or gene knockout experiments—are required to verify the predicted mechanisms.Despite these limitations, our findings provide a basis for further exploration of natural products in breast cancer treatment. Addressing these gaps would strengthen the mechanistic understanding and therapeutic potential of this extract.

## 6 Conclusion

Total saponins from *Ranunculus ternatus* Thunb. Can inhibit JAK2/STAT3 pathway activation; regulate upstream and downstream indicators; suppress BC cell growth, proliferation, and migration; promote cancer cell apoptosis; and reduce inflammatory responses, thereby exerting anti-BC effects.

## Data Availability

The original contributions presented in the study are publicly available. This data can be found here: [10.6084/m9.figshare.28812215].

## Abbreviations

Bcl-2, B-cell lymphoma-2; Bax, Bcl-2-associated X protein; GAPDH, glyceraldehyde-3-phosphate dehydrogenase; CCK-8, Cell Counting Kit-8; FBS, Fetal Bovine Serum; HE, Hematoxylin-Eosin Staining; IL, Interleukin; ICW, In-Cell Western; LSD, Least SignificantDifference; mRNA, messenger RNA; RSD, Relative Standard Deviation.
