# Supplementary material for: Mechanism of total saponins of Ranunculus ternatus Thunb. in treatment of breast cancer based on liquid chromatography–mass spectrometry and network analysis
Source: Front Pharmacol. 2025 Apr 25;16:1506885. doi: 10.3389/fphar.2025.1506885 (PMC12062126; doi:10.3389/fphar.2025.1506885)

**Supplementary material**

Figure 1: Raw data -- apoptotic flow diagram. The following are the apoptotic flow diagram of drug RRTS acting on breast cancer MCF-7 cells at 0 concentration, 50μg/mL concentration and 100μg/mL concentration respectively. The experiment was repeated three times, such as Test 1, Test 2 and Test 3.

Figure 1


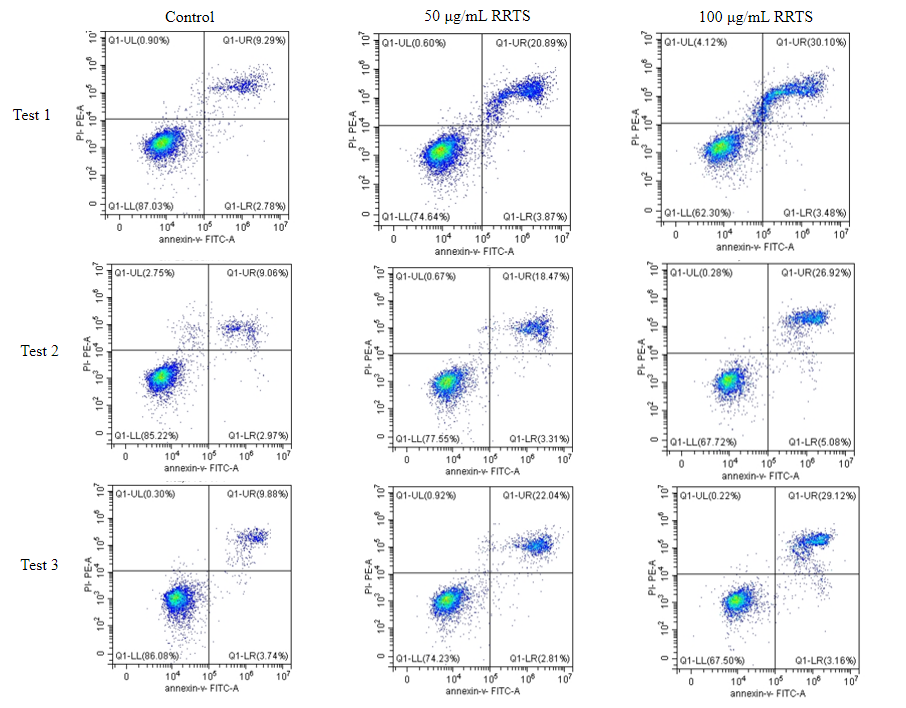


Figure 2 in cell experiment, the following are the in cell images of drug RRTS acting on breast cancer MCF-7 cells at 0 concentration, 50μg/mL concentration and 100μg/mL concentration respectively. Used to represent changes in JAK2, p-JAK2, STAT3, p-STAT3, Bax, Bcl-2 proteins, repeated three times, such as Test 1, Test 2, Test 3. Select Test 1 results to place in the article.

Figure 2


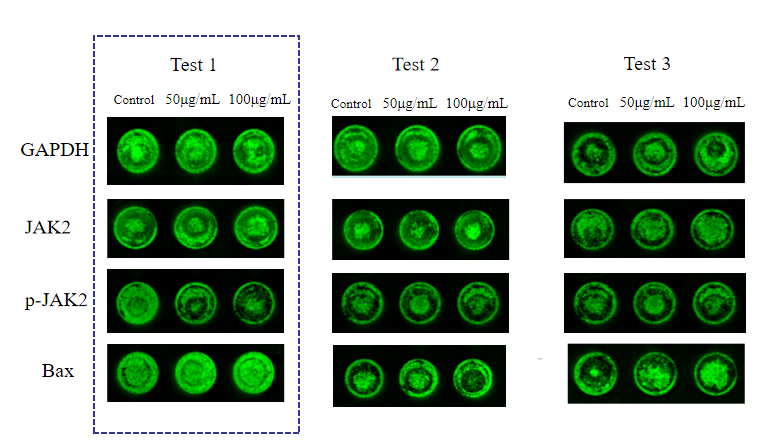


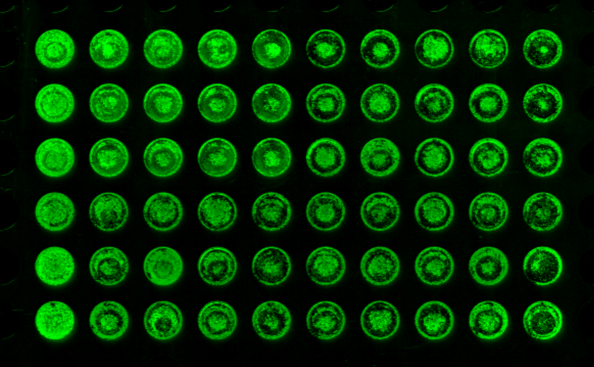


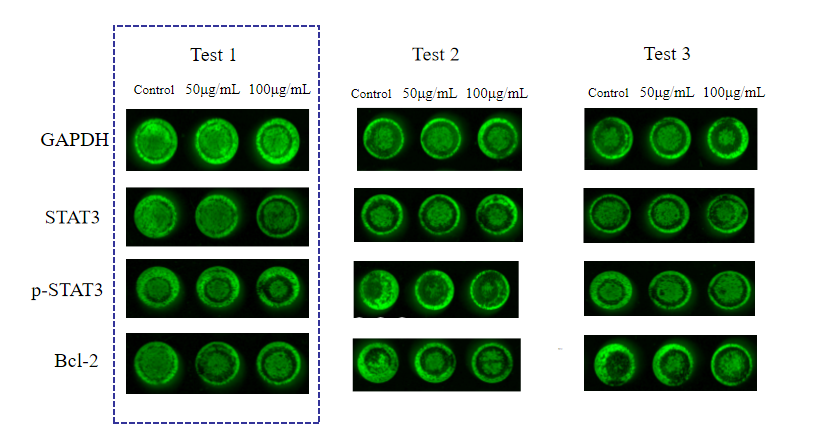

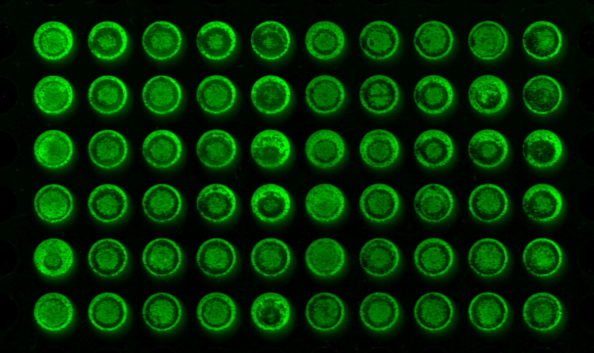

Supplement: Supplementary file 3 [file DataSheet1.docx]
